# Loss Separation Modeling and Optimization of Permalloy Sheets for Low-Noise Magnetic Shielding Devices

**DOI:** 10.3390/ma18194527

**Published:** 2025-09-29

**Authors:** Yuzheng Ma, Minxia Shi, Yachao Zhang, Teng Li, Yusen Li, Leran Zhang, Shuai Yuan

**Affiliations:** 1 Key Laboratory of Ultra-Weak Magnetic Field Measurement Technology, Ministry of Education, School of Instrumentation Science and Optoelectronics Engineering, Beihang University, Beijing 100191, China; 21myz@buaa.edu.cn (Y.M.); liysen@buaa.edu.cn (Y.L.); zhangleran@buaa.edu.cn (L.Z.); ys@buaa.edu.cn (S.Y.); 2 Beijing Institute of Aerospace Microsystem and Information Technology, Beijing 100094, China; simonazhyc@163.com

**Keywords:** SST measuring device, magnetic properties, hysteresis noise, Bertotti loss separation method, imaginary magnetic permeability

## Abstract

With the breakthroughs in quantum theory and the rapid advancement of quantum precision measurement sensor technologies, atomic magnetometers based on the spin-exchange relaxation-free (SERF) mechanism have played an increasingly important role in ultra-weak biomagnetic field detection, inertial navigation, and fundamental physics research. To achieve high-precision measurements, SERF magnetometers must operate in an extremely weak magnetic field environment, while the detection of ultra-weak magnetic signals relies on a low-noise background. Therefore, accurate measurement, modeling, and analysis of magnetic noise in shielding materials are of critical importance. In this study, the magnetic noise of permalloy sheets was modeled, separated, and analyzed based on their measured magnetic properties, providing essential theoretical and experimental support for magnetic noise evaluation in shielding devices. First, a single-sheet tester (SST) was modeled via finite element analysis to investigate magnetization uniformity, and its structure was optimized by adding a supporting connection plate. Second, an experimental platform was established to verify magnetization uniformity and to perform accurate low-frequency measurements of hysteresis loops under different frequencies and field amplitudes while ensuring measurement precision. Finally, the Bertotti loss separation method combined with a PSO optimization algorithm was employed to accurately fit and analyze the three types of losses, thereby enabling precise separation and calculation of hysteresis loss. This provides essential theoretical foundations and primary data for magnetic noise evaluation in shielding devices.

## 1. Introduction

With the rapid advancement of quantum theory and the continuous maturation of quantum sensor technologies [1,2], atomic magnetometers and inertial measurement devices based on the spin-exchange relaxation-free (SERF) [3,4] effect have played an increasingly important role in ultra-weak biomagnetic field measurement [5,6,7], inertial navigation, and fundamental physics research [8,9]. A low-noise [10,11], ultra-weak magnetic environment [12,13] is a prerequisite for atoms to enter the SERF [14] regime and to enable the detection of weak magnetic signals. Therefore, the design and construction of magnetically shielded rooms (MSRs) [15,16,17] are of critical importance, as they can effectively suppress the interference of external magnetic fields on experimental results and significantly enhance the reliability of research. The shielding of static and quasi-static magnetic fields in magnetically shielded rooms (MSRs) is primarily achieved through magnetic flux shunting by high-permeability, low-reluctance soft magnetic materials such as permalloy [18,19]. However, while permalloy exhibits these advantageous properties, it also possesses pronounced hysteresis and low electrical resistivity, which introduce significant noise. The noise level of permalloy imposes a limitation on the performance of high-precision atomic sensors [20,21]. Therefore, accurate calculation and evaluation of material-induced noise are essential for assessing the overall performance of MSRs.

Accurate evaluation of noise relies on precise magnetic property measurements [22]. At present, the most widely adopted methods for measuring the magnetic properties of electrical steel sheets are the Epstein frame method and the single-sheet tester (SST) method. The Epstein frame method [23], proposed in 1949, suffers from the disadvantages of complicated specimen preparation and the difficulty of determining the average magnetic path length, which inevitably affects measurement accuracy. In contrast, the single-sheet tester method avoids the unfavorable factor of calculating the effective magnetic path length. The International Electrotechnical Commission (IEC) has proposed the single sheet tester (SST), which has been repeatedly revised and is now regarded as the standard method for magnetic property characterization [24,25]. For permalloy sheets, the *B*–*H* sensing unit typically consists of a B-sensor and an H-coil, which enables high measurement accuracy without affecting the physical structure of the specimen [26]. The single sheet test method was first introduced in 1974 by the Japanese scholar Takaaki Yamamoto [27]. This approach directly measures the magnetic flux variation and magnetic field strength inside the specimen through *B*–*H* coils, thereby avoiding errors associated with effective magnetic circuit calculations. In 2009, Daisuke Miyagi and colleagues developed a novel single-sheet tester that enhanced the excitation field strength and reduced the overall size of the SST system to 60% of its original scale [28]. In 2022, Shi et al. introduced a negative feedback control system into the SST setup, further improving measurement accuracy [26]. Most recently, in 2024, Georgi et al. investigated the magnetization region, field homogeneity, and repeatability of specimens measured using the SST method [29,30].

The noise of permalloy primarily consists of hysteresis loss noise and eddy-current loss noise. The hysteresis loss noise is mainly related to the complex permeability and material thickness, whereas the eddy-current loss noise is associated with resistivity and material thickness. Kubo et al. first introduced the concept of thermal fluctuation–dissipation and, based on linear response theory, provided a proof of the fluctuation–dissipation theorem, thereby establishing a solid theoretical foundation for magnetic noise analysis in materials [31]. In 2013, the Italian National Institute of Metrological Research investigated a general method for calculating magnetic losses in soft magnetic composites (SMCs) under typical non-sinusoidal excitation conditions [32]. In 2022, Ma et al., based on hysteresis loss theory and loss separation theory, constructed a magnetic shielding barrel composed of ferrite and permalloy, and analyzed the calculation methods of both transverse and longitudinal magnetic noise [33]. More recently, in 2025, Zhao et al. proposed a magnetic noise modeling and suppression approach for shielding systems that takes into account residual loss noise and interlayer effects [34].

This paper investigates the low-frequency magnetic properties of permalloy measured using an optimized single-sheet tester (SST) device (Gopal Electronics, Ahmedabad, India) and applies the Bertotti loss separation model to decompose the various types of losses, thereby providing a theoretical foundation for the calculation of magnetic noise in magnetic shielding devices. First, the SST device was designed, constructed, and optimized. Subsequently, finite element simulations were performed to evaluate the magnetic field distribution of soft magnetic materials within the single-sheet testing setup, and the results were validated through experimental measurements of the actual magnetic field distribution, confirming the performance and reliability of the device. Finally, based on the low-frequency magnetic property data obtained from the measurements, the Bertotti loss separation model was employed to decompose the total loss into hysteresis loss, eddy current loss, and excess loss, followed by a detailed analysis of the loss distribution under low-frequency and weak-field conditions.

The remainder of this paper is organized as follows. Section 2 introduces the principle, structure, and optimization of the SST device, as well as finite element simulations of magnetization uniformity. Section 3 presents the experimental validation of the simulated uniformity and reports the measured low-frequency magnetic properties of permalloy. Section 4 focuses on the calculation, analysis, and separation of magnetic losses.

## 2. The Structure, Principle, Optimization, and Simulation of SST

**Figure 1:**
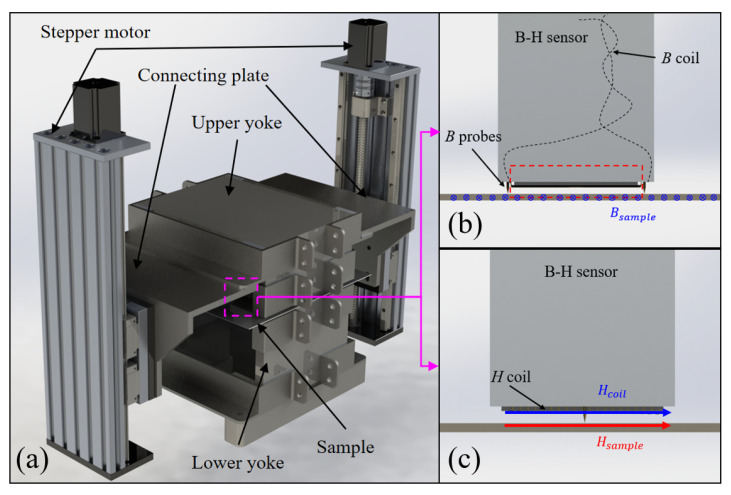
The SST structure: (**a**) overall sketch, (**b**) B measuring method, (**c**) H measuring method.

Figure 1a presents the structural arrangement of the SST apparatus. The excitation module is composed of two U-shaped magnetic yokes enclosing the driving coil. These yokes are fabricated from non-oriented silicon steel laminations in order to suppress eddy-current dissipation. In operation, the upper and lower yokes are aligned symmetrically with respect to the specimen, generating counter-acting magnetizing fields that facilitate a homogeneous distribution of magnetic flux.

The measurement configuration for the B–H characteristics incorporates a B-sensor, a sensing coil for flux density, and an H-coil. As illustrated in Figure 1b, the B-sensor, flux coil, and sample together establish a closed pathway for the magnetic flux density B. Upon magnetization of the sample, the flux coil produces an induced electromotive force, which, according to Faraday’s induction principle, can be transformed into the intrinsic magnetic flux density (*B_sample_*) of the material.

(1) $$B_{sample}=-\frac{1}{N_{B}S_{B}}\int V_{B}dt$$

where *V_B_* denotes the probe-induced electromotive force, *S_B_* corresponds to the cross-sectional region under measurement, *N_B_* indicates the effective coil turns, here approximated as 0.5.

The H-sensing coil in Figure 1c is mounted on a slender non-magnetic support and consists of several turns of insulated copper winding. It is positioned adjacent to the specimen surface. By enforcing the boundary continuity of the tangential *H* component between different media and invoking Faraday’s induction principle, the magnetic field strength within the specimen, *H_sample_*, can be calculated as

(2) $$H_{sample}=-\frac{1}{\mu_{0}N_{H}S_{H}}\int V_{H}dt$$

where *V_H_* denotes the electromotive force induced in the H-coil, *S_H_* is its cross-sectional area, *N_H_* refers to the coil winding number, and *μ*_0_ stands for the permeability of free space. Since the effective cross-section *S_H_* of the *H* coil, consisting of 1000 copper turns, varies with the winding count, calibration is required prior to measurement.

The conventional SST device applies an excitation magnetic field to permalloy sheets through a pair of magnetic yokes. The upper and lower yokes are aligned, and the specimen is clamped by the self-weight of the upper yoke. However, the self-weight introduces additional mechanical stress, to which permalloy is highly sensitive, thereby affecting the accuracy of magnetic property measurements. To eliminate this influence, the SST system was optimized and improved, as illustrated in Figure 1. Specifically, to prevent the direct load of the upper yoke on the specimen and the resulting distortion of its magnetic characteristics, the improved device rigidly connects the left and right support bases to the upper yoke using non-magnetic bolts. A motor-driven precision lead screw mechanism enables stepless adjustment of the support height, thereby allowing precise control of the gap between the yoke and the permalloy specimen. The yoke assembly adopts a U-shaped dual-yoke configuration, which ensures tight contact with the specimen and provides enhanced suppression of edge effects, thereby reducing magnetic flux leakage and improving field uniformity. Owing to the minimized edge effects, the dual-yoke structure can generate a more uniform magnetic field over a wider range, resulting in measurement outcomes that more closely approximate the intrinsic properties of the material.

**Figure 2:**
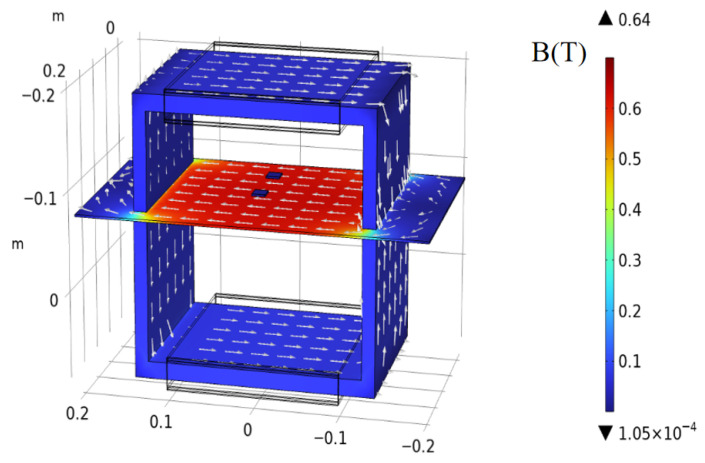
COMSOL simulation of the SST structure and the corresponding magnetic flux density *B* distribution.

To ensure that the measurement results accurately reflect the intrinsic magnetic properties of the material, it is also necessary to analyze the internal magnetic field distribution of the permalloy sheet during testing through electromagnetic simulation. In this work, a single-sheet measurement system was modeled using COMSOL Multiphysics 6.1, and an excitation current was applied to the excitation coil. The resulting distribution of the magnetic flux density *B* in the device is shown in Figure 2.

**Figure 3:**
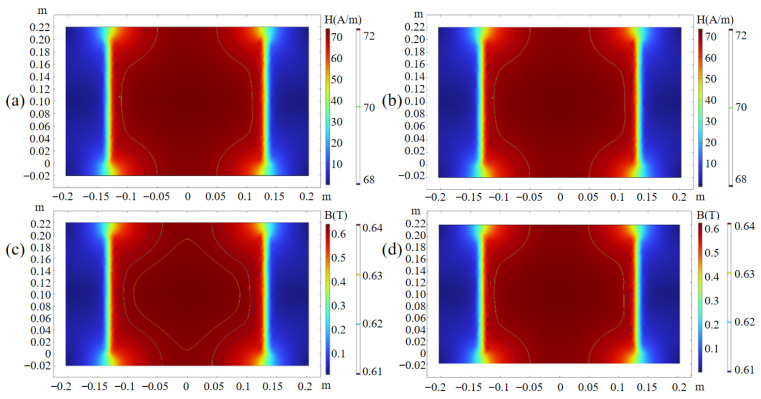
Simulation results of the magnetization distribution. (**a**) *H* on the supper surface. (**b**) *H* on the lower surface. (**c**) *B* on the upper surface. (**d**) *B* on the lower surface.

To evaluate whether the measurement results of the *B*–*H* sensor can accurately reflect the intrinsic magnetic properties of the material, both simulation and experimental analyses were conducted on the field uniformity within the specimen. The distributions of *B* and *H* on the upper and lower surfaces of the specimen are shown in Figure 3. The simulation results indicate that, within the central region of 120 mm × 140 mm, the deviations of the magnetic flux density *B* and the magnetic field strength *H* are both less than 2.86%, demonstrating that the magnetic field inside the specimen exhibits excellent uniformity.

To evaluate the effectiveness of the SST optimization via mechanical support to remove self-weight, repeated measurements of the hysteresis loops were performed at 17 Hz under *H* = 5 A/m and *H* = 9 A/m. Before optimization, the maximum deviations of *B* were 0.017 T and 0.0282 T for *H* = 5 A/m and *H* = 9 A/m, respectively. After optimization, the deviations decreased to 0.0018 T and 0.0058 T, representing a reduction by roughly one order of magnitude, which demonstrates the significant improvement in measurement consistency. The effect of the air gap between the yoke and the specimen on magnetization uniformity was evaluated through finite-element simulations. For a permalloy sheet in the central 120 mm × 140 mm region, air gaps of 0.2 mm, 0.4 mm, 1.0 mm, and 1.5 mm were considered. As the gap increased from 0.2 mm to 1.5 mm, the lower bound of flux density decreased from 0.2282 T to 0.2232 T (2.19%), and the upper bound decreased from 0.2327 T to 0.2278 T (2.11%). These results show that variations in air gap lead to only minor changes in magnetization uniformity and intensity, with fluctuations within 2%, indicating a negligible impact on measurement accuracy.

## 3. Experimental Validation of SST Magnetization Uniformity and Magnetic Property Measurement

**Figure 4:**
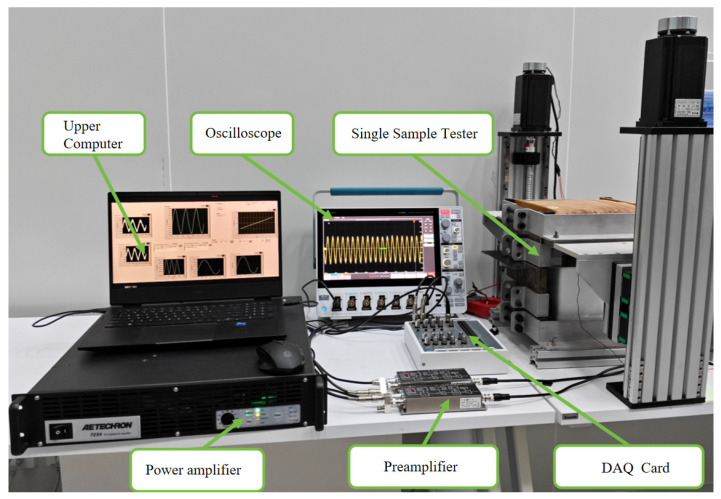
Experimental diagram of the SST measurement system.

Biomagnetic signals are extremely weak and mainly distributed in the low-frequency range, making accurate measurement and analysis dependent on high-performance magnetic shielding. Under low-frequency, low-magnetization excitation, *B*–*H* measurements can be affected by noise, reducing the signal-to-noise ratio and potentially impacting the precise evaluation of the shielding chamber’s performance. Therefore, a high-precision, low-noise preamplifier was employed. The SST sheet measurement setup is illustrated in Figure 4. The induced *B* and *H* voltages are first amplified using a FEMTO DLPVA-100 preamplifier (FEMTO Messtechnik GmbH, Berlin, Germany), which has a noise level of 700 pV/√Hz, and then digitized by an NI USB-6366 (National Instruments (NI), Austin, TX, USA) with 16-bit resolution before being transmitted to a host computer for post-processing. The excitation signal is supplied through an AE Techron 7234 power amplifier (AMETEK Electronic Instruments, Harrison, OH, USA) with a gain of 20.

**Figure 5:**
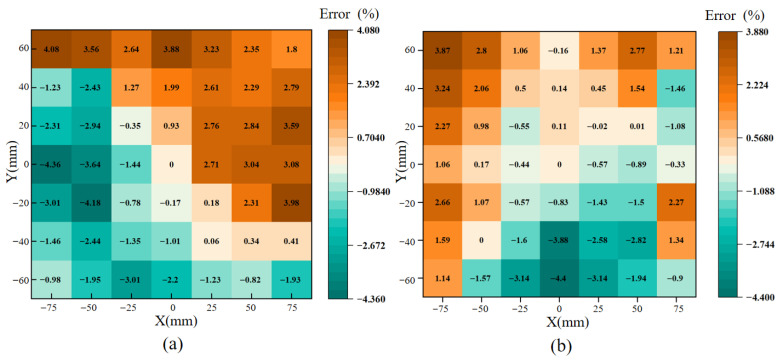
Experimental results of magnetization uniformity. (**a**) Upper surface. (**b**) Lower surface.

To verify whether the simulation results accurately reflect the magnetic field distribution characteristics of the actual measurement setup, the magnetic field strength at different positions on the upper and lower surfaces of the specimen was calculated from the induced voltage of the H coil. Based on these results, the surface magnetic field distribution was analyzed. The magnetic field strength measured at the center of the specimen was selected as the reference, and the deviations at other positions relative to the center were evaluated to assess the field uniformity within the target area of 120 mm × 140 mm, as shown in Figure 5.

The amplitude of the magnetic field strength at the center of the upper surface was 19.1251 A/m, with a maximum deviation of only 4.36% across the measurement area. The center value on the lower surface was 19.0549 A/m, which was essentially consistent with the upper surface, and the maximum deviation in this area was 4.40%. A comparison between simulation and experimental results showed that the relative errors of both upper and lower surfaces were small and remained below 5%, demonstrating that the simulation could reliably reproduce the magnetic field distribution of the specimen. The slightly larger deviations in measurement were mainly attributed to the non-ideal air gap between the magnetic yokes and the specimen, which induced localized leakage effects. Furthermore, within the practical measurement area of 50 mm × 50 mm at the specimen center, the maximum relative error on both surfaces was only 2.76%, with good repeatability. These results confirm that the designed measurement system provides high accuracy and can be effectively applied for the characterization of soft magnetic materials.

**Figure 6:**
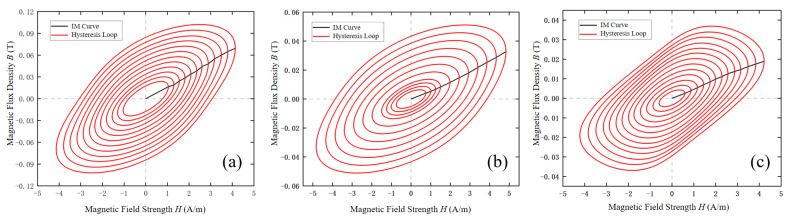
Experimental results of magnetic property measurements. (**a**) 11 Hz. (**b**) 57 Hz. (**c**) 69 Hz.

The SST applies forward excitation signals with varying amplitudes in the frequency range of 5–99 Hz to the permalloy specimen to obtain the corresponding magnetization curves. Figure 6 presents the measured hysteresis loops and initial magnetization curves (IC curve) of the specimen under increasing excitation fields at 11 Hz, 57 Hz, and 69 Hz. As shown, the area of the hysteresis loop expands with the increase in the magnetic field generated by the SST, indicating that the energy loss of the permalloy specimen grows during the magnetization process. Furthermore, when the magnetic field intensity is below 5 A/m at these three frequencies, the initial magnetization curve is nearly linear, suggesting that the material is in the initial magnetization region.

**Figure 7:**
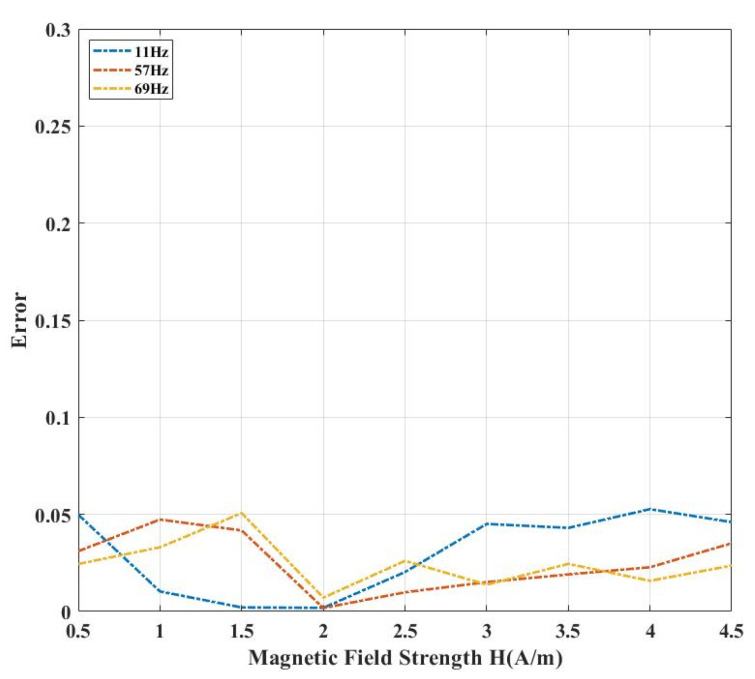
Repeatability of hysteresis loop measurements.

To ensure the accuracy and reliability of the measurements, error analysis and repeated measurements were conducted for the hysteresis loops at 11 Hz, 57 Hz, and 69 Hz shown in Figure 6. Specifically, for each frequency, hysteresis loops with magnetic field strengths from 0.5 A/m to 4.5 A/m were measured multiple times, and the maximum deviations from repeated measurements were analyzed, as shown in Figure 7. The results show that within this magnetization range, the maximum deviation did not exceed 5%, and most fluctuations remained between 2% and 3%, indicating that the experimental data exhibit good repeatability and measurement accuracy.

## 4. Principles and Experimental Analysis of Magnetic Losses

### 4.1. Principles of Magnetic Losses

The optimization design of a magnetically shielded room (MSR) must balance the dual requirements of high shielding efficiency and low intrinsic magnetic noise. Permalloy, as a representative shielding material, offers extremely high initial permeability that enables efficient attenuation of external magnetic fields. However, its high electrical conductivity gives rise to Johnson–Nyquist noise, while domain wall motion contributes to hysteresis and eddy-current losses. Together, these mechanisms dominate the low-frequency magnetic noise (0.1–100 Hz) of soft magnetic materials. In the low-frequency range (5–11 Hz), the frequency dependence of hysteresis loss is most pronounced, which is closely related to the dominant energy dissipation mechanism of soft magnetic materials under quasi-static magnetization. This frequency band not only encompasses the main components of biomagnetic signals (such as magnetocardiography and magnetoencephalography) but also overlaps significantly with the operating frequency range of SERF atomic magnetometers (0.1–100 Hz). Therefore, the shielding performance within this range directly determines the effectiveness of magnetic noise suppression and the accuracy of measurements. The experimental results of this study demonstrate that hysteresis loss makes the primary contribution in the 5–11 Hz range, providing important guidance for the optimization of shielding chamber design. Direct measurement of magnetic noise is challenging; therefore, this study employs an indirect characterization method based on the fluctuation–dissipation theorem. By combining hysteresis loop analysis with loss decomposition via the three-parameter Bertotti model, the influence of microstructural features (e.g., grain size, domain wall density) on magnetic noise can be quantitatively assessed. This provides theoretical guidance for optimizing low-noise shielding materials through treatments such as stress annealing or field-assisted thermal processing.

The magnetic noise at a given point can be inferred from the local power loss, while the total power loss of soft magnetic materials is obtained from hysteresis loops measured using a single-sheet tester. Since the hysteresis loop area directly represents the energy dissipation during magnetization, it serves as a practical basis for evaluating total power loss. Moreover, by applying Faraday’s law of electromagnetic induction and the principle of magnetic impedance, the induced electromotive force e can be quantitatively related to magnetic noise. The power loss per unit volume of a soft magnetic material over one cycle is expressed in Equation (3).

(3) $$\int e_{i}dt=\int H\frac{dB}{dt}dt=\int HdB$$

To accurately analyze the imaginary part of complex permeability relevant to magnetic noise, it is necessary to eliminate the coupling effects between eddy-current and excess losses through a loss-separation approach. The commonly used methods include: (1) hysteresis-loss modeling, where hysteresis loops are fitted to extract parameters, though with relatively high computational cost; (2) Steinmetz empirical formula, suitable for engineering estimation within specific frequency and flux density ranges, but lacking physical interpretation; and (3) Bertotti’s loss-separation method, which, based on domain-wall motion theory, decomposes the total loss (*P_e_*) into hysteresis loss (*P_hyst_*), classical eddy-current loss (*P_eddy_*), and excess loss (*P_exc_*).

Among these, Bertotti’s method is particularly advantageous due to its clear physical basis, broad frequency applicability, and strong relevance to processing conditions. In this study, it is therefore employed to separate the total loss measured by the single-sheet tester, enabling precise extraction of the hysteresis component that contributes to the imaginary part of permeability. According to this method, the total power loss of the material can be expressed as shown in Equation (4).

(4) $$P_{e}=P_{\mathrm{hyst}}+P_{eddy}+P_{exc}=(\varepsilon (B_{bias})k_{h}fB_{m}^{\lambda }+k_{e}f^{2}B_{m}^{2}+k_{exc}f^{1.5}B_{m}^{1.5})\rho_{e}V_{e}$$

where ε(*B_bias_*) = 1 + *k_dc_fB_m_^α^*, *ε*(*B_bias_*) represents the correction factor introduced by the DC magnetic field *B_bias_*. The coefficient *k_dc_* accounts for the DC contribution, and its measurement in magnetic shielding applications helps reduce the influence of the geomagnetic field on the total loss. In addition, *k_h_* denotes the hysteresis loss coefficient, reflecting the resistance to domain wall motion and strongly influenced by microstructural features such as grain size, grain boundary density, and annealing state. *λ* is the Steinmetz constant, which characterizes the nonlinearity of hysteresis loss with respect to magnetic induction. *k_e_* is the eddy-current loss coefficient, determined primarily by the material’s electrical conductivity and thickness. *k_exc_* represents the excess eddy-current loss coefficient associated with additional dissipation mechanisms induced by microstructural imperfections, such as impurities, defects, and grain boundary discontinuities. *B_m_* is the amplitude of the magnetic flux density, *ρ_e_* is the density of the tested specimen, and *V_e_* is its volume.

From the measurement results, the power losses under different frequencies and magnetic field strengths were calculated. By applying the Bertotti loss separation method, the hysteresis loss coefficient (*k_h_*), the eddy-current loss coefficient (*k_e_*), and the excess eddy-current loss coefficient (*k_exc_*) were fitted, thereby enabling the quantification of the three loss components. The hysteresis loss derived from the coefficient *k_h_* was further used to calculate the imaginary part of the complex permeability according to Equation (5), providing a high-accuracy dataset for modeling hysteresis noise in magnetic shielding barrels and supporting the noise-optimized design of multilayer permalloy structures. Since the power loss of high-permeability shielding materials is dominated by hysteresis and eddy-current components, Equations (5) and (6) can be employed to calculate and represent the corresponding hysteresis noise and eddy-current noise in magnetic shielding devices.

(5) $$P_{hyst}=\int_{V}\frac{1}{2}\omega \mu^{\prime \prime }H^{2}dv$$

(6) $$P_{eddy}=\int_{V}\frac{1}{2}\sigma E^{2}dv$$

### 4.2. Calculation and Analysis of Loss Separation

**Figure 8:**
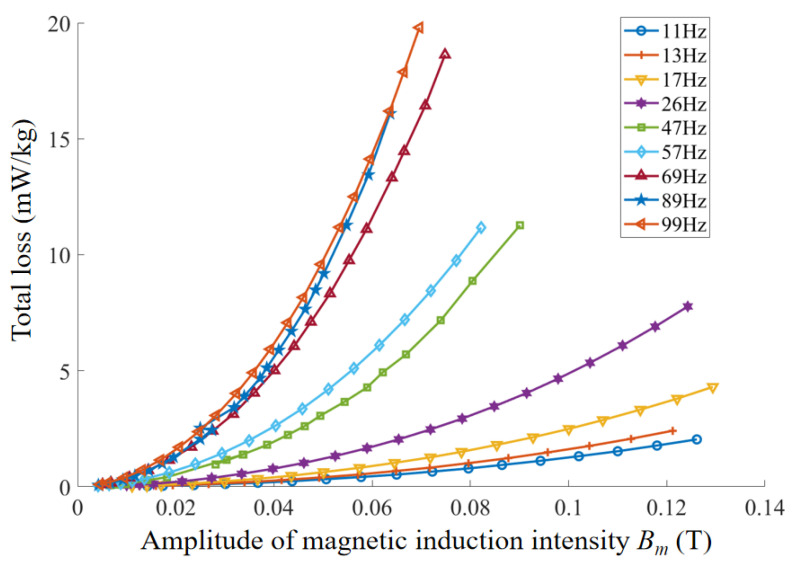
Calculated loss characteristics of permalloy.

By integrating the hysteresis loops under different frequencies and magnetic field excitations, the total magnetic loss of the material under each operating condition can be accurately calculated. The loss distribution of the in-furnace permalloy cylinder specimen is shown in Figure 8.

As observed, within the frequency range of 5–99 Hz, the total loss of the permalloy specimen increases with the amplitude of magnetic induction B, and the rate of increase becomes faster at higher frequencies. At a fixed magnetic induction, the total loss of the specimen also increases with frequency. When the magnetic induction is below 0.06 T, the total material loss remains less than 20 mW/kg across all frequencies. Since the extraction of the imaginary part of the complex permeability requires precise separation of the hysteresis loss, this study employs the Bertotti loss separation model to fit the coefficients and quantify hysteresis loss, eddy current loss, and excess loss. By establishing the functional relationships of these loss components with frequency and magnetic induction, the underlying loss mechanisms are further elucidated, thereby providing data support for magnetic noise evaluation of magnetic shielding cylinders.

**Figure 9:**
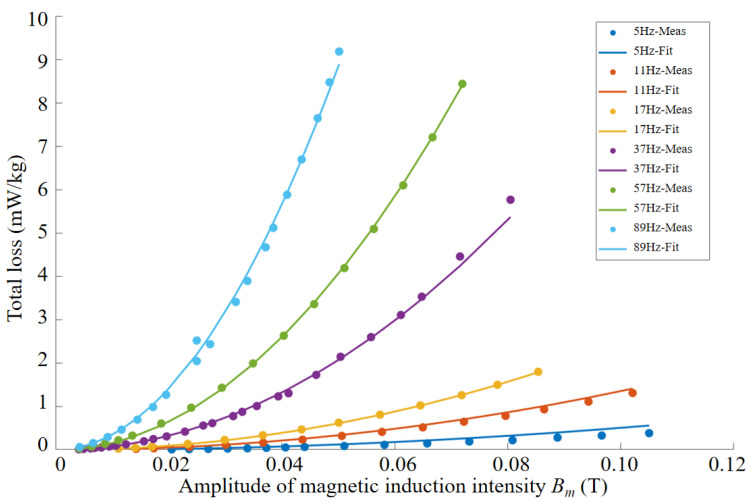
Loss measurement and fitting results.

To separate the hysteresis loss from the total loss of the material, this study employs a multi-parameter nonlinear empirical model to fit the loss characteristics of the permalloy specimen. The fitted parameters yield a Steinmetz coefficient *λ* of 2, a hysteresis loss coefficient *k_h_* = 0.008493, an eddy current loss coefficient *k_e_* = 0.0003511, and an excess eddy current loss coefficient *k_exc_* = 1.184 × 10^−7^. The fitting results are shown in Figure 9, with a coefficient of determination *R*^2^ = 0.9966, and a sum of squared errors (SSE) of 3.021 × 10^−5^, indicating that the original data closely match the model predictions and thus exhibit excellent predictive performance. The proposed loss separation model is established on the basis of experimentally measured magnetic properties and is, therefore, generally applicable to permalloy specimens with different geometries and thicknesses once the measurement data are determined.

In addition to biomagnetic measurements such as magnetoencephalography (MEG) and magnetocardiography (MCG), the implications of magnetic shielding loss and noise analysis are also highly relevant to transcranial magnetic stimulation (TMS). TMS involves the generation of strong pulsed magnetic fields, which impose stringent requirements on shielding performance and noise evaluation. The separation and quantification of hysteresis, eddy-current, and excess losses presented in this work provide a theoretical framework that can be extended to assess the magnetic noise induced by shielding materials in TMS environments, thereby facilitating the translation of our results into practical biomedical applications.

## 5. Conclusions

In this work, the magnetic noise of permalloy sheets was accurately extracted, separated, and analyzed based on magnetic property measurements and the Bertotti loss separation method, with further calculation and modeling of the imaginary part of the complex permeability. Firstly, a stepper motor and supporting plate were introduced to eliminate the stress induced by the self-weight of the upper yoke, thereby minimizing its influence on the magnetic properties. Finite element simulations were then conducted to evaluate the field uniformity within the central measurement region of the SST device. Secondly, experimental validation confirmed that the maximum fluctuation of magnetic field intensity was 4.36% within the central 120 mm × 140 mm area, and only 2.76% within the central 50 mm × 50 mm area. Using the high-precision SST system, hysteresis loops were measured and analyzed over the frequency range of 5–99 Hz under different excitation amplitudes. Finally, the total loss was calculated based on the measured data, and the Bertotti loss separation method was employed for parameter fitting, achieving precise separation of hysteresis loss, eddy current loss, and excess loss. The fitting results yielded a coefficient of determination *R*^2^ of 0.9966 and an SSE of 3.021 × 10^−5^, demonstrating excellent fitting accuracy. This precise loss separation provides a solid theoretical foundation and reliable data support for further analysis of the hysteresis characteristics of permalloy and for magnetic noise evaluation in shielding devices.

## Data Availability

The original contributions presented in the study are included in the article. Further inquiries can be directed to the corresponding authors.
